# 0026. Voluntary activation of the sympathetic nervous system and attenuation of the innate immune response in humans

**DOI:** 10.1186/2197-425X-2-S1-O2

**Published:** 2014-09-26

**Authors:** M Kox, LT van Eijk, J Zwaag, J van den Wildenberg, FCJG Sweep, JG van der Hoeven, P Pickkers

**Affiliations:** Radboud University Medical Center, Intensive Care Medicine, Nijmegen, Netherlands; Radboud University Medical Center, Anesthesiology, Nijmegen Netherlands; Nijmegen Institute for Infection, Inflammation and Immunity, Nijmegen, Netherlands; Radboud University Medical Center, Laboratory Medicine, Nijmegen, Netherlands

## Introduction

Excessive or persistent pro-inflammatory cytokine production plays a central role in a variety of inflammatory conditions. Acute activation of the sympathetic nervous system attenuates innate immunity. However, both the autonomic nervous system and innate immune system are regarded as systems that cannot be voluntarily influenced.

## Objectives

To evaluate the effects of a training program on the autonomic nervous system and innate immune response.

## Methods

We performed a parallel randomized controlled study in healthy male volunteers. Subjects were randomized to receive either a 10-day training program involving meditation (third eye meditation), breathing techniques (i.a., cyclic hyperventilation followed by breath retention), and exposure to cold (i.a., immersions in ice cold water), or no training. Subjects in both groups (n=12 per group) underwent experimental human endotoxemia (intravenous administration of 2 ng/kg *E. Coli* lipopolysaccharide [LPS]) during which the trained individuals practiced the learned techniques.

## Results

**Figure 1:**
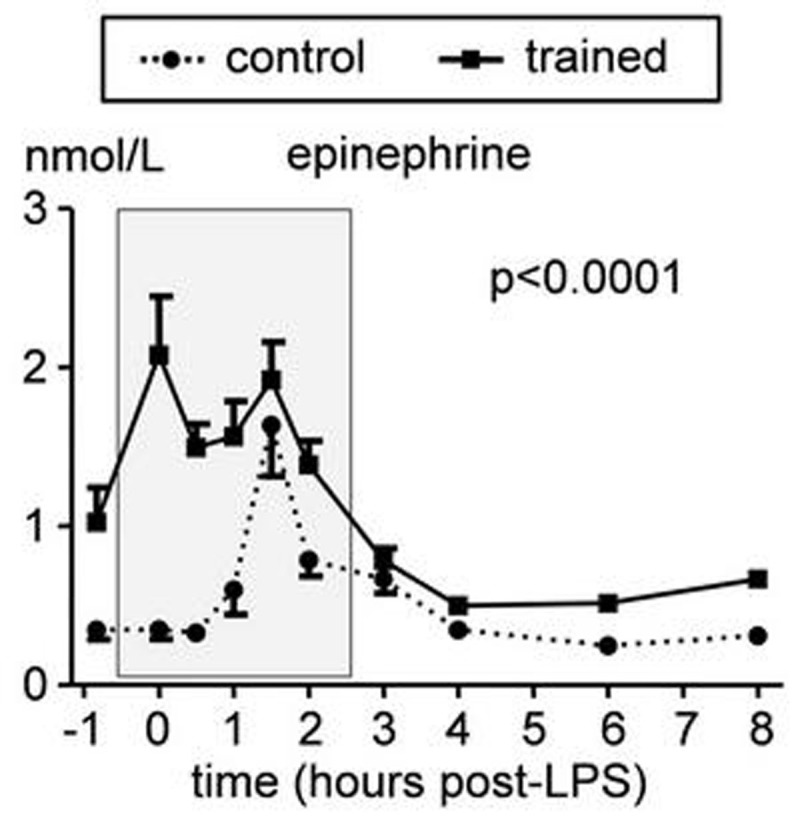
Plasma epinephrine concentrations during experimental endotoxemia in control and trained subjects. Data are expressed as mean ± SEM of 12 subjects per group. Gray box indicates period in which the trained subjects practiced their learned breathing techniques. P values between groups were calculated using repeated measures two-way analysis of variance (ANOVA, interaction term).

**Figure 2:**
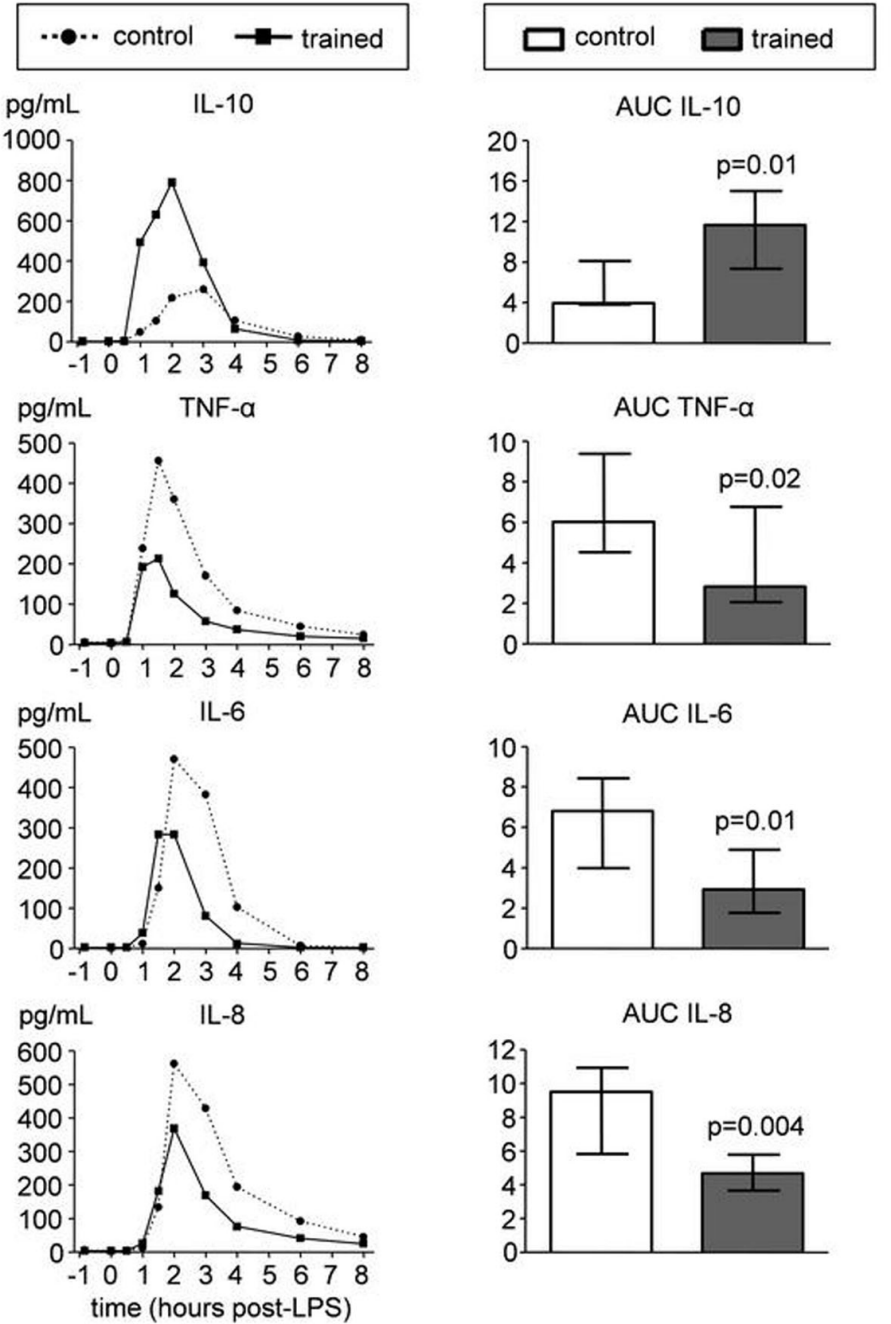
Plasma cytokine concentrations during endotoxemia in control (n=12) and trained (n=12) subjects. Left panels depict median values of anti-(IL-10) and pro-inflammatory (TNF-α, IL-6, and IL-8) cytokines. Right panels depict median ± interquartile range of area under curve (AUC) of cytokines (unit:×10^4^ pg/mL·h). P values were calculated using Mann-Whitney U-tests.

Practicing the learned techniques resulted in intermittent respiratory alkalosis and hypoxia resulting in significantly increased plasma epinephrine levels (Figure 1). In the trained group, plasma levels of the anti-inflammatory cytokine IL-10 increased more rapidly after LPS administration, correlated strongly with preceding epinephrine levels (r=0.82, p=0.001), and were higher (Figure 2). Levels of pro-inflammatory mediators TNF-α, IL-6, and IL-8 were lower in the trained group (Figure 2) and correlated negatively with IL-10 levels (r=-0.71, p=0.01; r=-0.59, p=0.045; r=-0.71, p=0.01, respectively). Finally, LPS-induced flu-like symptoms and fever were blunted in the trained group.

## Conclusions

Voluntary activation of the sympathetic nervous system results in epinephrine release and subsequent suppression of the innate immune response in humans *in vivo*. These results could have important implications for the treatment of a variety of conditions associated with excessive or persistent inflammation.

